# Antiretroviral Treatment Gaps and Adherence Among People with HIV in the U.S. Medicare Program

**DOI:** 10.1007/s10461-023-04208-8

**Published:** 2023-10-27

**Authors:** Pengxiang Li, Girish Prajapati, Zhi Geng, Vrushabh P. Ladage, Jean Marie Arduino, Dovie L. Watson, Robert Gross, Jalpa A. Doshi

**Affiliations:** 1 University of Pennsylvania, Philadelphia, PA USA; 2 Merck & Co., Inc., Rahway, NJ USA

**Keywords:** Medicare, HIV, Adherence, Treatment gaps, Discontinuation, Antiretrovirals, Claims

## Abstract

Approximately one-quarter of people with HIV (PWH) in the U.S. receive coverage through the Medicare program; however, no prior real-world study has examined antiretroviral therapy (ART) gaps and adherence and associated factors in this population. This retrospective cohort analysis used 2013–2018 national Medicare fee-for-service claims data to identify all PWH initiated on a new ART regimen including protease inhibitors [PI], non-nucleoside reverse transcriptase inhibitors [NNRTIs], or integrase strand transfer inhibitors [INSTIs] between 1/1/2014 and 12/31/2017. Study outcomes included ART adherence (based on proportion of days covered [PDC]), continuous treatment gaps ranging from 1 to 6 days to ≥ 180 days, and discontinuation (continuous gap ≥ 90 days) in the 12-month follow-up period. Multivariable regressions were used to assess factors associated with ART adherence and discontinuation. The final sample included 48,627 PWH (mean age: 54.5 years, 74.4% male, 47.5% White, 89.8% disabled). Approximately 53.0% of PWH had a PDC ≥ 0.95, 30.2% had a PDC between 0.70 and < 0.95, and 16.8% had PDC < 0.70. Treatment gaps of at least ≥ 7-days (55.2%) and ≥ 30-days (26.2%) were common and 10.1% PWH discontinued treatment. Younger age, female sex, Black race, higher comorbidity score, mental health conditions, and substance use disorder were associated with higher odds of lower adherence and discontinuation (all p-values < 0.05). In conclusion, suboptimal adherence and treatment gaps in ART use were commonly observed among PWH in Medicare. Interventions and policies to mitigate barriers to adherence are urgently needed in this population to both improve their survival and increase the potential for ending the HIV epidemic in the US.

**Supplementary Information:**

The online version contains supplementary material available at 10.1007/s10461-023-04208-8.

## Introduction

There are approximately 1.1 million people with HIV (PWH) in the United States (U.S.), with over 30,000 newly-diagnosed cases in 2020 [1]. The U.S. Medicare program—federally-funded health insurance for elderly (≥ 65 years) adults and younger patients with certain disabilities—has become a major source of funding for HIV care. Since the 1990s, the number of Medicare beneficiaries with HIV has tripled [2]. Most Medicare beneficiaries with HIV are eligible for the Medicare program due to disability; however, the number of elderly PWH has steadily increased [2]. Approximately one-quarter of PWH receive health insurance coverage through Medicare, and the program is now the largest source of federal financing for HIV care and treatment [2]. Furthermore, nearly 300,000 PWH will reach age 65 by 2030 and become eligible for the Medicare program [1].

Like all PWH, Medicare beneficiaries have benefitted from the advent of antiretroviral therapy (ART), which revolutionized HIV treatment and significantly lowered mortality and morbidity rates [3, 4]. PWH are expected to continue ART treatment indefinitely. Thus, ART adherence is critical for PWH, given its importance in achieving durable virologic suppression and minimizing drug resistance [4, 5]. However, ART regimens differ in dosing frequency, complexity, and tolerability, which cumulatively influence optimal adherence and treatment outcomes [6–10].

A recent systematic literature review of HIV medication adherence studies did not identify a single study analyzing empirical claims-based data of ART adherence or treatment gaps among Medicare beneficiaries with HIV [11]. This is a critical gap in the literature given the unique circumstances that may impact medication adherence among Medicare beneficiaries including polypharmacy and high comorbidity burden. The objective of this study was to examine ART treatment patterns (adherence, gaps in treatment, and discontinuation) and assess associated factors among PWH in the Medicare program.

## Methods

### Study Design and Data Source

This was a retrospective analysis using 2013–2018 Chronic Conditions Warehouse (CCW) 100% Medicare Part A, B, and D claims available from the Centers for Medicare and Medicaid Services (CMS) in the U.S. Our data extract included the CCW 100% Medicare files for all fee-for-service Medicare beneficiaries with an HIV diagnosis between 2013 and 2018. The Medicare Part A and B files include claims for inpatient care, skilled nursing facility care, home health services, outpatient services, durable medical equipment, and hospice services covered by the fee-for-service Medicare program. The Part D files contain all outpatient prescription drug events covered by Part D and include information on the date of fill, the National Drug Code that identifies generic/brand name and prescription strength, the number of days’ supply, the quantity of pills dispensed, and costs associated with each prescription. The Medicare claims files are linked to Medicare personal summary files, which contain information on enrollees’ demographics, eligibility, and date of death, if applicable.

### Study Sample

**Fig. 1:**
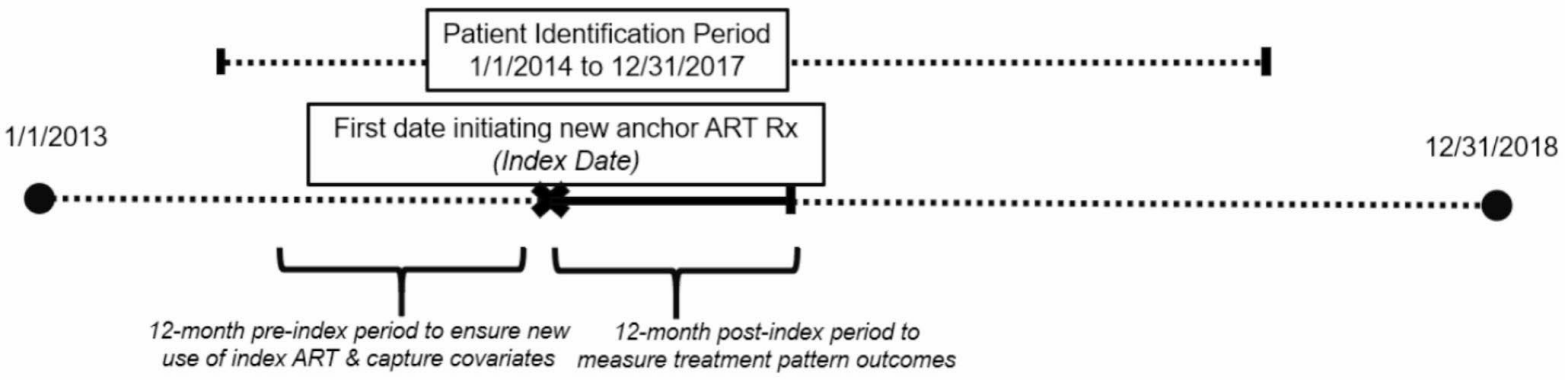
Sample selection schematic

Figure 1 presents the sample selection schematic. We identified all PWH who initiated a new ART regimen between 01/01/2014 and 12/31/2017 including either treatment-naïve PWH who initiated ART or treatment-experienced PWH who switched to a new ART regimen. We categorized ART regimens by class of new “anchor medication” initiated: protease inhibitors (PIs), non-nucleoside reverse transcriptase inhibitors (NNRTIs), or integrase strand transfer inhibitors (INSTIs). Anchor medications were identified from fixed-dose combination single-tablet regimens (STR) or multi-tablet regimens (MTR, **Electronic Supplement** Table 1). The fill date of the first prescription of the new anchor medication was deemed the index date. PWH were required to meet the following inclusion criteria: (1) evidence of ≥ 1 prescription claim for a new anchor medication between 01/01/2014 to 12/31/2017, (2) continuous eligibility for fee-for-service Medicare Parts A, B, and D covered in the 12 months pre- and post-index period, and (3) ≥ 1 inpatient or outpatient claim with an HIV diagnosis in the 12-month pre-index period (ICD-9-CM codes 042, V08, 079.53 or ICD-10-CM codes B20, B97.35, O98.711, O98.712, O98.713, O98.719, O98.72, O98.73, Z21) [12–14]. PWH were excluded if they: (1) died during the 12-month post-index period, (2) were missing values on key covariates (i.e., age, sex, identification code for county of residence), and/or (3) were started on multiple anchor medications on the index date.

**Table 1:** Demographic and clinical characteristics of people with HIV in the U.S. Medicare program who initiated a new index anchor agent

	N	%
N	48,627	100.0
Age group, years		
≤34	2,122	4.4
35–44	5,729	11.8
45–54	16,930	34.8
55–64	15,182	31.2
65–74	7,076	14.6
75–84	1,497	3.1
≥85	91	0.2
Sex		
Male	36,164	74.4
Female	12,463	25.6
Race/ethnicity*		
White	23,079	47.5
Black	21,052	43.3
Hispanic	3,044	6.3
Other	1,452	3.0
Low-income subsidy (LIS) status		
Full LIS	39,944	82.1
Non-LIS	7,685	15.8
Partial LIS	998	2.1
Original reason for Medicare eligibility		
Age	4,456	9.2
Disability**	43,640	89.8
ESRD**	1,677	3.5
Census region (%)		
Midwest	6,326	13.0
Northeast	10,765	22.1
South	22,949	47.2
West	8,587	17.6
Metropolitan status***		
Rural	4,498	9.3
Urban	44,129	90.7
Medicare Part D plan type		
Enhanced alternative Part D plan	8,154	16.8
Others	40,427	83.2
Year of the index date		
2014	18,730	38.5
2015	11,615	23.9
2016	11,332	23.3
2017	6,950	14.3
Drug class & STR status of Index anchor ART		
INSTI and STR	18,003	37.0
INSTI and MTR	14,748	30.3
NNRTI and STR	4,927	10.1
NNRTI and MTR	2,915	6.0
PI	8,034	16.5
CMS-HCC risk score, mean (SD)	48,627	1.04 (0.92)
Comorbidities****		
Cardiovascular conditions and risk factors	34,280	70.5
Renal disease	10,795	22.2
Liver diseases	8,782	18.1
Cancer (non-AIDS and AIDS-defining cancer)	4,032	8.3
Mental health conditions	18,052	37.1
Substance use disorder	14,827	30.5
Lung diseases	10,146	20.9
Autoimmune conditions	11,210	23.1
Endocrine disorders	1,957	4.0
Gastrointestinal disorders	12,920	26.6
Number of 30-day supply prescriptions filled per month in the pre-index period, mean (SD)	48,627	6.10 (4.37)

Abbreviations: ART, antiretroviral therapy; CMS-HCC risk score: Centers for Medicare and Medicaid Services Hierarchical Condition Categories risk score; ESRD, end-stage-renal disease; INSTI, Integrase Strand Transfer Inhibitor; MTR: multi-tablet regimen; NNRTI, Non-Nucleoside Reverse Transcriptase Inhibitor; PI, Protease Inhibitor; SD: standard deviation; STR: single-tablet regimen

*Medicare administrative data does not have separate fields for race and ethnicity. We are unable to differentiate ethnicity within race (e.g., Hispanic Black vs. non-Hispanic Black). Beneficiaries who were reported as a race or ethnicity other than White, Black, or Hispanic were consolidated into the “Other” category and include Asian, North American Native, and Other

**Includes 1146 people with both disability and ESRD; ***Beneficiary level rurality categories defined using Rural-Urban Commuting Area codes and categorized as urban (RUCA 1.0 - 3.0) and rural (RUCA 4.0 - 10.3).

****Categories for the comorbidities included the following:

*Cardiovascular conditions and risk factors* include acute myocardial infarction; cardiac arrhythmias, ischemic heart disease, stroke/transient ischemic attack, heart failure, and peripheral vascular disease, hyperlipidemia, hypertension, obesity, overweight, and diabetes mellitus

*Liver diseases* include cirrhosis, chronic hepatitis B, non-alcoholic fatty liver disease or non-alcoholic steatohepatitis, and chronic hepatitis C

*Mental health conditions* include depression, anxiety disorders, bipolar or manic depression, trauma-stressor related disorders, and suicidal ideation

*Substance use* include drug use disorders, alcohol use disorders, opioid use disorder, and tobacco use disorder

*Lung diseases* include chronic obstructive pulmonary disease and asthma

*Autoimmune conditions* include rheumatoid arthritis, multiple sclerosis, inflammatory bowel diseases, psoriasis, psoriatic arthritis, systemic lupus erythematosus, and hypothyroidism

*Gastrointestinal disorders* include diarrhea, peptic ulcer disease, nausea or vomiting, and esophageal reflux

### Outcomes

HIV treatment guidelines in the U.S. recommend a combination of three antiretroviral medications with two drugs from the NRTI class and a third “anchor” medication from the PI, NNRTI, or INSTI class. Given the highly correlated nature of adherence to the anchor medication with the NRTI medications in a regimen, we focused the adherence outcome measure on the third “anchor” medications. This approach was adapted from Hines et al. (2019) [15]. Adherence to anchor medications was measured using prescription fill data, including the date of fill and days’ supply reported on each prescription claim. Adherence to anchor medications in the 12-month post-index period was examined using proportion of days covered (PDC) defined as the number of days covered by an anchor medication divided by the number of days in the period (i.e., 365 days from the index date) [16]. For example, if 292 days were covered by an anchor medication during the 12-month post-index period, then the beneficiary had a PDC of 0.80 (292 ÷ 365). To account for the possibility that an individual switched anchor medications during the 12-month post-index period (and would thus inappropriately be classified as “poorly adherent”), we measured adherence to any anchor medication rather than adherence to the index anchor medication. Days spent in the hospital, skilled nursing facility, or hospice were excluded from both the denominator and numerator because the individual might have received medications dispensed from the facility’s pharmacy rather than the beneficiary’s own supply. If supplies for the anchor medication overlapped with days spent in a facility, we adjusted the prescription start date to be the day after the stay ended to account for possible stockpiling [17]. If the same anchor ART prescription was filled before the end of day supply of the last fill, the medication supply was carried over to adjust for each day of overlap. For example, if an individual filled drug A with 30-day supply on day 1 and obtained a second fill for the same drug A on the 28th day, we adjusted the fill date for the second prescription from the 28th day to the 31st day. Optimal adherence to any anchor medication was defined as a PDC ≥ 0.95 based on existing clinical HIV treatment guidelines and the criteria most widely used in the HIV literature [9, 13, 18, 19]. Additionally, we measured adherence using different thresholds (PDC ≥ 0.70, PDC ≥ 0.80, and PDC ≥ 0.90) to account for previous studies that found lower PDC levels might be sufficient to achieve viral suppression [11, 20, 21]. Adherence patterns among beneficiaries were grouped into three PDC categories for our main analysis: (1) PDC ≥ 0.95, (2) 0.95 > PDC ≥ 0.70, (3) PDC < 0.70.

Treatment gap in the use of anchor medications was a dichotomous variable, defined as the presence of a fixed period with no supply of an anchor medication after the days’ supply of the most recent anchor prescription was exhausted. To account for possible anchor medication switches during the follow-up period, we coded continuous gaps in the use of any anchor medications (defined as the number of days without any anchor medication supply) and discontinuation of all anchor medications (defined as a continuous 90-day gap without any anchor medication supply). To examine treatment gaps, we spread the reported days’ supply from each prescription for an anchor medication from the fill date to the date the supply would have been exhausted in the follow up period. We then calculated whether each day in the post-index period was covered (or not covered) by an anchor medication’s days’ supply to identify gaps in any anchor medication use. Any day the beneficiary spent in the hospital, skilled nursing facility, or hospice was not counted as a gap day in the gap measurement for similar reasons as stated earlier for the adherence measure. We categorized the duration of each continuous treatment gap into one of eight categories: <7, 7–13, 14–29, 30–89, 90–119, 120–149, 150–179, or ≥ 180 days. We measured the percentage of beneficiaries with at least one gap and the total number of gaps per person in the 12-month post-index period. ART discontinuation was a dichotomous variable, defined as the presence of a continuous treatment gap of ≥ 90 days [14, 15, 22]. Time to discontinuation was a continuous variable, defined as the number of days from the index date to the date of discontinuation (i.e., beginning of ≥ 90-day continuous treatment gap).

### Analysis

Descriptive statistics were calculated to describe the demographic and clinical characteristics of the sample. Multivariable regression models were used to examine factors associated with anchor medication adherence and discontinuation. Multinomial logistic regression was used to examine the categorical PDC adherence outcome (PDC ≥ 0.95, 0.95 > PDC ≥ 0.70, and PDC < 0.70). Logistic regression was used for the binary outcome of anchor medication discontinuation (continuous gap of ≥ 90 days or not). Multivariable Cox regression was used to examine factors associated with time to discontinuation. All multivariable regressions included sociodemographic and clinical covariates selected based on prior literature and clinical expert inputs. The covariates included age group, sex, race/ethnicity, receipt of Part D low income subsidy (LIS), original reason for Medicare eligibility, U.S. census region, metropolitan status, Part D plan type, year of the index date, index anchor medication class by STR status, CMS Hierarchical Condition Category (CMS-HCC) risk score [23], presence of selected comorbidities, and number of prescriptions filled per month in the pre-index period.

All analyses were performed using SAS Enterprise Guide Version 9.4 (SAS Institute, Cary, NC). This study was deemed exempt from review by the Institutional Review Board of the University of Pennsylvania.

## Results

The final study sample included 48,627 PWH who initiated a new anchor medication between 01/01/2014 and 12/31/2017 (**Electronic Supplement** Table 2). Mean age was 54.5 years (SD: 10.9); 82.2% were younger than 65 years old; and 74.4% were male (Table 1). Based on race/ethnicity categories available in the data, 47.5% were White, 43.3% were Black, 6.3% were Hispanic, and 3% were another race/ethnicity. Most beneficiaries were eligible for Medicare due to disability (89.8%) and qualified for full Part D low-income subsidies (82.1%). Nearly half (47.2%) resided in the South census region, and 90.7% resided in an urban area. The index anchor medication for the majority of PWH enrolled in Medicare was an INSTI (67.3% overall; 37.0% STR and 30.3% MTR), followed by a PI (16.5% overall), and an NNRTI (16.1% overall; 10.1% STR and 6.0% MTR). Most beneficiaries (70.5%) had cardiovascular conditions and risk factors, 37.1% had a mental health condition, and 30.5% had a substance use disorder. PWH enrolled in Medicare experienced a high medication burden (including non-ART medications) with a mean of 6.1 prescriptions (SD 4.4) filled per month.

**Table 2:** Descriptive outcomes of adherence and treatment gap among people with HIV in the U.S. Medicare program, overall and by class of index anchor agent

	Overall		INSTI		NNRTI		PI	
	N	%	N	%	N	%	N	%
N	48,627	100.0	32,751	100.0	7842	100.0	8034	100.0
**Adherence Measures**								
PDC, mean (SD)	48,627	0.86 (0.21)	32,751	0.87 (0.20)	7842	0.85 (0.22)	8034	0.81 (0.25)
**Adherence cut-offs**								
PDC ≥ 0.95	25,760	53.0	18,011	55.0	4124	52.6	3625	45.1
PDC ≥ 0.90	31,806	65.4	22,111	67.5	5109	65.1	4586	57.1
PDC ≥ 0.80	37,380	76.9	25,842	78.9	6015	76.7	5523	68.7
PDC ≥ 0.70	40,456	83.2	27,830	85.0	6,513	83.1	6,113	76.1
**Adherence groups**								
PDC < 0.70	8171	16.8	4921	15.0	1329	16.9	1921	23.9
PDC ≥ 0.70 to < 0.95	14,696	30.2	9819	30.0	2389	30.6	2488	31.0
PDC ≥ 0.95	25,760	53.0	18,011	55.0	4124	52.6	3625	45.1
**Treatment Gap Measures**								
Longest continuous gap								
0 days (no gap)	9363	19.3	6695	20.4	1448	18.5	1220	15.2
1 to 6 days	12,408	25.5	8523	26.0	2045	26.1	1840	22.9
7 to < 14 days	6899	14.2	4764	14.5	1079	13.8	1056	13.1
14 to < 30 days	7208	14.8	4931	15.1	1149	14.7	1128	14.0
30 to < 90 days	7839	16.1	5016	15.3	1260	16.1	1563	19.5
90 to < 120 days	1427	2.9	858	2.6	230	2.9	339	4.2
120 to < 150 days	935	1.9	572	1.7	142	1.8	221	2.8
150 to < 180 days	669	1.4	380	1.2	124	1.6	165	2.1
≥180 days	1879	3.9	1012	3.1	365	4.7	502	6.2
Discontinuation (gap ≥90 days)	4910	10.1	2822	8.6	861	11.0	1227	15.3
Time to discontinuation from index date (in days)								
Mean [SD]	118.2	[77.9]	123.4	[78.1]	109.2	[76.9]	112.5	[77.0]
Median [IQR]	106.0	[30.0, 183.0]	115.0	[39.0, 188.0]	93.0	[30.0, 167.0]	95.0	[30.0, 178.5]

Abbreviations: INSTI, Integrase Strand Transfer Inhibitor; IQR: interquartile range; NNRTI, Non-Nucleoside Reverse Transcriptase Inhibitor; PDC: proportion of days covered; PI, Protease Inhibitor; SD: standard deviation

Table 2 presents the adherence and treatment gaps observed in our sample, overall and by anchor medication class. The mean PDC was 0.86 for any anchor medication (Table 2). More than half of enrollees (53.0%) had a PDC ≥ 0.95, 30.2% had a PDC between 0.70 and < 0.95 and 16.8% had PDC < 0.70. PWH initiating an index anchor medication from the INSTI class had the highest PDC (0.87), followed by those initiating NNRTI-based regimens (0.85) and PI-based regimens (0.81). At least one continuous treatment gap of 7 days or more was common (55.2%) and over one-quarter (26.2%) of the sample had a continuous gap of 30 days or more. One in ten PWH enrolled in Medicare (10.1%) had evidence of ART discontinuation. The median time to discontinuation was 106 days (IQR 30–183). Discontinuation rates were lowest among PWH who initiated INSTI-based regimens (8.6%) and highest among those who initiated PI-based regimens (15.3%).

Several factors were associated with lower adherence and discontinuation in the multivariate analyses. The multinomial logit regressions showed that younger age, female sex, Black race, Hispanic ethnicity, full low-income subsidy status, higher comorbidity score, index anchor medication from the PI class, and comorbidities such as a mental health condition or substance use disorder were associated with statistically significant higher odds of being in the lower adherence groups relative to the PDC ≥ 0.95 group (Table 3). The factors associated with any anchor ART discontinuation identified from the binomial logistic regressions were quite similar. For example, younger age (age ≤ 34 years: OR 1.54, 95% CI: 1.30–1.83; age 35–44: OR 1.54, 95% CI: 1.32–1.79 vs. age 65–74), female sex (OR 1.20, 95% CI: 1.12–1.29), Black race (OR 1.17, 95% CI: 1.08–1.26 vs. White race), higher comorbidity score (OR 1.08, 95% CI: 1.03–1.13), and comorbidities such as a mental health condition (OR 1.16, 95% CI: 1.08–1.25) or substance use disorder (OR 1.32, 95% CI: 1.23–1.42) were associated with statistically significant higher odds of any anchor ART discontinuation (Table 4). PWH who initiated an INSTI-based regimen (STR or MTR) and those who initiated an NNRTI-based STR had lower odds of treatment discontinuation compared to those who initiated a PI-based regimen. Multivariable Cox regressions showed similar results in terms of factors associated with time to treatment discontinuation.

**Table 3:** Factors associated with different adherence levels on any anchor ART agent over 12-months of follow-up from initiation of a new anchor ART agent* among people with HIV in the U.S. Medicare program

	PDC ≥ 0.70 to < 0.95 vs. PDC ≥ 0.95						PDC < 0.70 vs. PDC ≥ 0.95				
	OR	95% CI		Chi-Square	P value		OR	95% CI		Chi-Square	P value
**Age group**											
≤ 34	1.18	1.03	1.35	5.83	0.016		1.86	1.58	2.19	56.66	< 0.001
35–44	1.28	1.16	1.41	23.33	< 0.001		1.91	1.67	2.18	89.10	< 0.001
45–54	1.18	1.08	1.27	15.17	< 0.001		1.4	1.24	1.58	29.68	< 0.001
55–64	1.02	0.94	1.11	0.28	0.597		1.11	0.98	1.25	2.61	0.106
65–74 (Reference)											
75–84	1.13	0.99	1.29	3.12	0.077		1.13	0.92	1.4	1.31	0.253
≥ 85	1.54	0.97	2.46	3.35	0.067		1.32	0.63	2.74	0.55	0.46
**Sex**											
Male (Reference)											
Female	1.06	1.01	1.11	4.66	0.031		1.23	1.15	1.31	39.17	< 0.001
Race/ethnicity**											
White (Reference)											
Black	1.32	1.25	1.38	120.47	< 0.001		1.49	1.4	1.59	143.28	< 0.001
Hispanic	1.15	1.05	1.25	8.71	0.003		1.14	1.01	1.29	4.51	0.034
Other	0.94	0.83	1.07	0.93	0.334		1.02	0.86	1.21	0.06	0.807
**Low-income subsidy (LIS) status**											
Full LIS	1.14	1.06	1.21	14.23	< 0.001		1.27	1.15	1.39	23.70	< 0.001
Partial LIS	1.07	0.92	1.25	0.80	0.371		0.95	0.75	1.19	0.24	0.627
Non-LIS (Reference)											
**Original reason for Medicare eligibility**											
Age (Reference)											
Disability	1.16	1.05	1.28	9.19	0.002		1.23	1.08	1.42	8.86	0.003
ESRD	1.37	1.20	1.56	21.62	< 0.001		1.93	1.65	2.27	64.75	< 0.001
**Census region**											
Northeast (Reference)											
South	1.05	0.99	1.11	2.56	0.110		1.14	1.06	1.23	12.35	< 0.001
Midwest	0.96	0.9	1.04	1.00	0.318		0.93	0.84	1.03	1.89	0.170
West	0.93	0.87	0.99	4.58	0.032		1.02	0.92	1.12	0.11	0.742
**Metropolitan status**											
Rural	1.12	1.04	1.21	9.51	0.002		1.07	0.97	1.18	2.01	0.156
Urban (Reference)											
**Medicare Part D plan type**											
Enhanced alternative plan type	1.00	0.94	1.06	0.02	0.902		0.88	0.81	0.96	7.75	0.005
Others (Reference)											
**Year of the index date**											
2014 (Reference)											
2015	0.98	0.93	1.04	0.43	0.510		0.80	0.74	0.86	37.20	< 0.001
2016	0.84	0.78	0.9	25.37	< 0.001		0.57	0.52	0.62	144.37	< 0.001
2017	0.79	0.73	0.86	34.27	< 0.001		0.54	0.49	0.60	128.65	< 0.001
**Index anchor ART drug class**											
INSTI and STR	0.78	0.73	0.83	53.78	< 0.001		0.56	0.51	0.60	188.52	< 0.001
INSTI and MTR	0.89	0.83	0.95	12.48	< 0.001		0.77	0.71	0.84	39.05	< 0.001
NNRTI and STR	0.80	0.73	0.87	27.41	< 0.001		0.57	0.51	0.63	107.07	< 0.001
NNRTI and MTR	1.04	0.94	1.15	0.54	0.464		1.08	0.94	1.23	1.22	0.270
PI (Reference)											
**CMS-HCC risk score**	1.06	1.03	1.09	12.83	< 0.001		1.11	1.07	1.16	25.84	< 0.001
Comorbidities***											
Cardiovascular and cerebrovascular conditions	0.91	0.87	0.96	12.42	< 0.001		1.00	0.94	1.06	0.01	0.925
Renal disease: chronic kidney disease, end-stage-renal disease	1.06	1.00	1.12	3.45	0.063		1.23	1.13	1.33	25.01	< 0.001
Liver diseases	1.09	1.03	1.15	8.91	0.003		1.04	0.96	1.12	0.91	0.341
Cancer: non-AIDS and AIDS-defining cancer	1.00	0.92	1.08	0.01	0.915		0.97	0.87	1.08	0.30	0.581
Mental health conditions	1.22	1.16	1.28	66.40	< 0.001		1.31	1.22	1.39	65.78	< 0.001
Substance use disorder	1.25	1.19	1.32	79.29	< 0.001		1.59	1.49	1.69	198.34	< 0.001
Lung diseases	1.10	1.04	1.16	10.18	0.001		1.30	1.21	1.40	49.10	< 0.001
Autoimmune conditions	1.09	1.04	1.15	11.00	0.001		1.29	1.20	1.39	45.97	< 0.001
Endocrine disorders	0.99	0.89	1.10	0.05	0.82		0.78	0.66	0.93	7.50	0.006
GI disorders	1.13	1.07	1.18	20.30	< 0.001		1.31	1.22	1.40	59.83	< 0.001
**Number of 30-day supply prescriptions filled per month in the pre-index period**	0.89	0.88	0.90	1366.43	< 0.001		0.70	0.69	0.71	4184.18	< 0.001

Abbreviations: AIDS: acquired immunodeficiency syndrome; ART: antiretroviral therapy; CI: confidence interval; CMS-HCC risk score: Centers for Medicare and Medicaid Services Hierarchical Condition Categories risk score; ESRD: end-stage renal disease; GI: gastrointestinal disorders; INSTI: Integrase Strand Transfer Inhibitor; MTR: multi-tablet regimen; NNRTI: Non-Nucleoside Reverse Transcriptase Inhibitor; OR: odds ratio; PDC: proportion of days covered; PI: Protease Inhibitor; STR: single-tablet regimen

*Multinomial logit on three categories of outcome (PDC < 0.70 and 0.70 ≤ PDC < 0.95 versus PDC ≥ 0.95)

**Medicare administrative data does not have separate fields for race and ethnicity. We are unable to differentiate ethnicity within race (e.g., Hispanic Black vs. non-Hispanic Black). Beneficiaries who were reported as a race or ethnicity other than White, Black, or Hispanic were consolidated into the “Other” category and include Asian, North American Native, and Other

***Categories for the comorbidities included the following:

*Cardiovascular conditions and risk factors* include acute myocardial infarction; cardiac arrhythmias, ischemic heart disease, stroke/transient ischemic attack, heart failure, and peripheral vascular disease, hyperlipidemia, hypertension, obesity, overweight, and diabetes mellitus

*Liver diseases* include cirrhosis, chronic hepatitis B, non-alcoholic fatty liver disease or non-alcoholic steatohepatitis, and chronic hepatitis C

*Mental health conditions* include depression, anxiety disorders, bipolar or manic depression, trauma-stressor related disorders, and suicidal ideation

*Substance use* include drug use disorders, alcohol use disorders, opioid use disorder, and tobacco use disorder

*Lung diseases* include chronic obstructive pulmonary disease and asthma

*Autoimmune conditions* include rheumatoid arthritis, multiple sclerosis, inflammatory bowel diseases, psoriasis, psoriatic arthritis, systemic lupus erythematosus, and hypothyroidism

*Gastrointestinal disorders* include diarrhea, peptic ulcer disease, nausea or vomiting, and esophageal reflux

**Table 4:** Factors associated with anchor ART discontinuation (90-day gap) and time to anchor ART discontinuation over 12-months of follow-up from initiation of new anchor ART agent among people with HIV in the U.S. Medicare program*

Outcome	Discontinuation (90-day gap)						Time to discontinuation (90-day gap)				
	OR	0.95% CI		Chi-Square	P value		HR	0.95% CI		Chi-Square	P value
**Age group**											
≤ 34	1.54	1.30	1.83	24.16	< 0.001		1.43	1.22	1.66	20.66	< 0.001
35–44	1.54	1.32	1.79	31.49	< 0.001		1.46	1.27	1.67	28.88	< 0.001
45–54	1.13	0.98	1.3	2.86	0.091		1.12	0.98	1.27	2.77	0.096
55–64	0.96	0.84	1.11	0.26	0.610		0.97	0.85	1.11	0.22	0.641
65–74 (Reference)											
75–84	0.98	0.76	1.25	0.04	0.847		0.98	0.77	1.23	0.04	0.833
≥ 85	1.49	0.72	3.09	1.13	0.289		1.39	0.72	2.71	0.96	0.327
**Sex**											
Male (Reference)											
Female	1.20	1.12	1.29	25.62	< 0.001		1.18	1.11	1.26	27.32	< 0.001
Race/ethnicity**											
White (Reference)											
Black	1.17	1.08	1.26	16.72	< 0.001		1.16	1.08	1.24	18.86	< 0.001
Hispanic	1.04	0.90	1.19	0.23	0.633		1.04	0.91	1.18	0.30	0.587
Other	1.04	0.85	1.26	0.13	0.724		1.02	0.85	1.22	0.04	0.834
**Low-income subsidy (LIS) status**											
Full-LIS	1.07	0.97	1.20	1.71	0.192		1.07	0.97	1.18	1.93	0.164
Partial-LIS	0.81	0.62	1.06	2.33	0.127		0.81	0.63	1.04	2.68	0.102
Non-LIS (Reference)											
**Original reason for Medicare eligibility**											
Age (Reference)											
Disability	1.06	0.91	1.24	0.51	0.476		1.04	0.90	1.20	0.34	0.561
ESRD	1.36	1.14	1.62	11.80	0.001		1.30	1.11	1.51	11.08	0.001
**Census region**											
Northeast (Reference)											
South	1.15	1.06	1.26	10.85	0.001		1.14	1.05	1.23	10.65	0.001
Midwest	0.99	0.88	1.11	0.05	0.82		0.99	0.89	1.1	0.04	0.838
West	1.06	0.95	1.18	1.01	0.315		1.06	0.96	1.17	1.23	0.267
**Metropolitan status**											
Rural	1.03	0.92	1.15	0.27	0.602		1.03	0.93	1.13	0.29	0.593
Urban (Reference)											
**Medicare Part D plan type**											
Enhanced alternative plan type	0.87	0.79	0.97	6.56	0.01		0.88	0.8	0.97	7.04	0.008
Others (Reference)											
**Year of the index date**											
2014 (Reference)											
2015	0.82	0.76	0.89	22.56	< 0.001		0.83	0.78	0.90	24.39	< 0.001
2016	0.61	0.55	0.68	86.18	< 0.001		0.64	0.58	0.71	83.50	< 0.001
2017	0.60	0.53	0.68	68.97	< 0.001		0.63	0.56	0.70	68.30	< 0.001
**Drug class & STR status of Index anchor ART**											
INSTI and STR	0.59	0.54	0.64	129.72	< 0.001		0.62	0.57	0.67	130.59	< 0.001
INSTI and MTR	0.79	0.72	0.86	28.60	< 0.001		0.82	0.76	0.88	25.86	< 0.001
NNRTI and STR	0.71	0.64	0.80	33.01	< 0.001		0.75	0.67	0.82	32.46	< 0.001
NNRTI and MTR	1.02	0.89	1.18	0.09	0.764		1.05	0.92	1.19	0.54	0.464
PI (Reference)											
**CMS-HCC risk score (continuous variable)**	1.08	1.03	1.13	10.81	0.001		1.06	1.02	1.11	10.24	0.001
Comorbidities***											
Cardiovascular conditions and risk factors	1.01	0.94	1.08	0.03	0.866		1.01	0.95	1.07	0.05	0.817
Renal disease: chronic kidney disease, end-stage-renal disease	1.15	1.05	1.26	8.84	0.003		1.12	1.03	1.22	7.55	0.006
Liver diseases	0.95	0.87	1.04	1.15	0.283		0.96	0.89	1.04	1.02	0.312
Cancer non-AIDS and AIDS-defining cancer	0.90	0.79	1.01	3.08	0.079		0.90	0.81	1.01	3.28	0.070
Mental health conditions	1.16	1.08	1.25	16.75	< 0.001		1.14	1.06	1.21	14.91	< 0.001
Substance use disorder	1.32	1.23	1.42	58.54	< 0.001		1.28	1.2	1.36	56.38	< 0.001
Lung diseases	1.15	1.06	1.25	11.21	0.001		1.12	1.05	1.21	9.95	0.002
Autoimmune conditions	1.2	1.10	1.30	17.02	< 0.001		1.16	1.08	1.26	15.40	< 0.001
Endocrine disorders	0.80	0.65	0.99	4.05	0.044		0.82	0.67	1.00	3.67	0.055
Gastrointestinal disorders	1.13	1.05	1.22	9.62	0.002		1.11	1.04	1.19	8.77	0.003
**Number of 30-day supply prescriptions filled per month in the pre-index period**	0.74	0.73	0.75	2098.47	< 0.001		0.76	0.75	0.76	2210.31	< 0.001

Abbreviations: AIDS: acquired immunodeficiency syndrome; ART: antiretroviral therapy; CI: confidence interval; CMS-HCC risk score: Centers for Medicare and Medicaid Services Hierarchical Condition Categories risk score; ESRD: end-stage renal disease; GI: gastrointestinal disorders; INSTI: Integrase Strand Transfer Inhibitor; MTR: multi-tablet regimen; NNRTI: Non-Nucleoside Reverse Transcriptase Inhibitor; OR: odds ratio; HR: hazard ratio; PDC: proportion of days covered; PI: Protease Inhibitor; STR: single-tablet regimen

*Binomial logit regression for discontinuation outcome and Cox regression for time to discontinuation outcome

**Medicare administrative data does not have separate fields for race and ethnicity. We are unable to differentiate ethnicity within race (e.g., Hispanic Black vs. non-Hispanic Black). Beneficiaries who were reported as a race or ethnicity other than White, Black, or Hispanic were consolidated into the “Other” category and include Asian, North American Native, and Other

***Categories for the comorbidities included the following:

*Cardiovascular conditions and risk factors* include acute myocardial infarction; cardiac arrhythmias, ischemic heart disease, stroke/transient ischemic attack, heart failure, and peripheral vascular disease, hyperlipidemia, hypertension, obesity, overweight, and diabetes mellitus

*Liver diseases* include cirrhosis, chronic hepatitis B, non-alcoholic fatty liver disease or non-alcoholic steatohepatitis, and chronic hepatitis C

*Mental health conditions* include depression, anxiety disorders, bipolar or manic depression, trauma-stressor related disorders, and suicidal ideation

*Substance use* include drug use disorders, alcohol use disorders, opioid use disorder, and tobacco use disorder

*Lung diseases* include chronic obstructive pulmonary disease and asthma

*Autoimmune conditions* include rheumatoid arthritis, multiple sclerosis, inflammatory bowel diseases, psoriasis, psoriatic arthritis, systemic lupus erythematosus, and hypothyroidism

*Gastrointestinal disorders* include diarrhea, peptic ulcer disease, nausea or vomiting, and esophageal reflux

## Discussion

To our knowledge, this is the first study to examine ART treatment patterns and associated factors among PWH enrolled in the Medicare program. As such, it offers critical insights on ART adherence and potential subpopulations to prioritize in ongoing efforts to provide high quality HIV care in this understudied but growing segment of the U.S. population.

Our study found a mean PDC of 0.86 and optimal adherence (PDC ≥ 0.95) among 53% of beneficiaries with HIV during the follow-up period. This is notably higher than previous studies among commercially-insured PWH, which have found optimal adherence levels of approximately 40% or less [24, 25]. Although our findings suggest better adherence among PWH in the Medicare program compared to the commercially-insured population, 1 in 6 Medicare beneficiaries (16.8%) had suboptimal adherence with a PDC < 0.70. Additionally, ART treatment gaps were common: 1 in 4 beneficiaries experienced treatment gaps of 30 days or more and 1 in 10 beneficiaries discontinued treatment over 12 months of follow-up. It should be noted that while our study assessed adherence and treatment gaps over a 12-month period after ART initiation, these medications must be taken indefinitely to achieve viral suppression, maintain healthy immune function, prevent antiretroviral resistance, and reduce HIV transmission. Hence, any evidence of suboptimal adherence or ART discontinuation are concerning. These concerns are compounded by the fact that Medicare beneficiaries with HIV have been shown to have higher mortality rates compared to beneficiaries without HIV [26]. Therefore, maintaining optimal ART adherence and reducing gaps in treatment among beneficiaries with HIV as they age and develop other chronic conditions is critical to improving health outcomes. Providers should work with patients to identify opportunities and resources to support adherence and avoid ART discontinuation.

Several factors were associated with suboptimal treatment adherence and discontinuation. Younger PWH were less likely to be adherent compared to PWH over the age of 65, underscoring the need for age-specific strategies to improve adherence among younger beneficiaries [27]. Consistent with prior literature in the US, female PWH in our study were more likely to be in the lower adherence groups and more likely to discontinue ART compared to males [14, 19, 28]. Additionally, our study joins the wide body of literature demonstrating persistent inequities in HIV care continuum outcomes among Black and Hispanic PWH [29]. We found that odds of optimal ART adherence were significantly higher among White PWH relative to Black and Hispanic PWH. These inequities are largely driven by systematic barriers that have been articulated elsewhere, including racism, unemployment, structural poverty, stigma, low literacy, homelessness, and homophobia [30, 31]. Interventions at system and structural levels are needed to address the needs of these populations and achieve equitable HIV care outcomes.

We found that PWH receiving a Part D low-income subsidy were less likely to be in the optimal adherence group. This finding may seem somewhat counterintuitive given high out-of-pocket costs are known to be a barrier to ART adherence and yet PWH receiving a low-income subsidy are theoretically shielded from such costs since they are only required to pay fixed nominal copayments per prescription. However, given the high monthly medication burden observed in these PWH it is possible that even small copayments add up to be unaffordable for these vulnerable patients. Prior work has shown that compared to White Medicare beneficiaries, substantially larger proportions of Black and Hispanic Medicare beneficiaries are eligible for Part D low-income subsidies due to low income and minimal assets and report problems paying medical bills, delaying care due to cost, and debt to collection agencies due to medical bills [32]. Therefore, LIS status in our analysis likely serves as a surrogate marker of severe financial burden and social determinants of health more broadly. Suboptimal ART adherence found among these groups is likely driven by underlying structural inequities and disparate experiences across multiple social determinants of health rather than the out-of-pocket costs alone. Future research should investigate multilevel approaches to support ART adherence and reduce discontinuation by addressing the needs of LIS Medicare beneficiaries.

Relative to PWH whose index anchor ART was a PI, those on INSTI-based regimens (MTR or STR) and NNRTI-based STR had lower odds of being in the suboptimal adherence and ART discontinuation groups. These findings are consistent with a previous analysis of commercial claims data, wherein authors found that PI-based regimens demonstrated a greater risk of discontinuation relative to INSTI-based regimens and NNRTI-based STR [14]. One explanation for our finding is that unlike NNRTIs and INSTIs, PIs were not available as a fixed-dose STR until 2018 (i.e. until after our sample identification window). In post-hoc analyses stratified by index anchor medication class, we found that STR users had lower odds of having suboptimal adherence and discontinuation relative to MTR users, among subgroups of patients on NNRTI- and INSTI-based regimens (**Electronic Supplement** Table 3). Similar findings were confirmed in a meta-analysis, which found that STRs have several advantages over MTRs for HIV treatment in terms of adherence, therapy continuation, viral suppression, tolerability, and quality of life [33]. At the same time, additional post-hoc analysis in the subgroup of MTR users found that patients on PI-based regimens had higher odds of suboptimal adherence relative to NNRTI- and INSTI-based regimens (**Electronic Supplement** Table 3). Hence, another possible explanation is the higher risk of drug-drug interactions, inhibitor-induced metabolic syndromes (e.g., dyslipidemia, insulin resistance), and cardiovascular events (e.g., myocardial infarction, ischemic stroke) associated with PI-based regimens [34, 35]. It is likely that the nearly 1 in 6 PWH (16.5%) in our sample who were started on a PI-based regimen may have done so due to prior suboptimal ART adherence and/or documented resistance to NNRTI- or INSTI-based regimens. Future work is needed to examine trends in PI use in more recent years and the reasons for prescribing PI-based regimens among PWH with Medicare.

Interestingly, fewer than 1 in 5 beneficiaries in our sample were prescribed a single-tablet regimen. This represents substantially lower use of STRs compared to the commercially insured population: a prior study by Kangethe et al. (2019) found that 41.2% of commercially-insured PWH used an STR [12]. Access barriers to STRs are unlikely to explain these findings since HIV drugs are a protected drug class in the Medicare Part D program, wherein all drugs must be covered on the plan formulary [36]. Also, most PWH in our sample (82%) were eligible for a full low-income subsidy with fixed nominal copayments *per prescription* and hence STRs would result in lower out-of-pocket costs than MTRs. Given the high burden of chronic medical conditions, it is possible that beneficiaries with HIV were prescribed MTRs rather than fixed-dosed STRs to adjust each antiretroviral for renal insufficiency, hepatic impairment, or potential drug-drug interactions [37].

Finally, we found several comorbidities to be associated with suboptimal adherence or discontinuation. Most importantly, PWH with mental health conditions or substance use disorders were more likely to be in the suboptimal adherence and discontinuation groups. These findings are consistent with prior published literature and suggest that tailored interventions to support these groups are needed in the Medicare program [14, 28].

Our study has several limitations. First, our findings are based on a national sample of fee-for-service Medicare beneficiaries with Part D plan coverage and hence may not be generalizable to individuals in Medicare Advantage plans or other types of insurance (e.g., commercial). Second, as with all claims-based studies, prescription fills do not account for whether the medication dispensed was taken as prescribed, potentially overestimating adherence and underestimating discontinuation. Third, we were unable to determine whether those who discontinued treatment eventually restarted after the 12-month follow-up period ended; therefore, we may have overestimated ART discontinuation. Fourth, potential reasons for suboptimal adherence or treatment gaps including side effects, out-of-pocket costs, patient preferences, or use as post-exposure prophylaxis (PEP) therapy were not available in our administrative claims data. Thus, we were unable to determine if treatment gaps were deliberate and appropriate. Nevertheless, ART is recommended for all persons with HIV in the U.S. to reduce morbidity and mortality and to prevent the transmission of HIV; therefore, we would expect all Medicare beneficiaries with HIV to receive treatment. Finally, unobserved or unmeasured variables may have confounded the relationship observed between measured factors and our study outcomes of adherence and discontinuation.

Despite these limitations, our study provides insights into an important but understudied group of Medicare beneficiaries with HIV and has direct implications for clinical practice and policy. Among fee-for-service Medicare beneficiaries with HIV who initiated a new anchor medication between 2014 and 2017, suboptimal adherence and gaps in continuous ART use were commonly observed. Our findings provide insights into the characteristics of PWH with suboptimal ART adherence and those discontinuing ART treatments. Interventions and policies to mitigate barriers to adherence are urgently needed to achieve more equitable HIV care continuum outcomes among Medicare beneficiaries, particularly those who are younger, Black, have a history of a mental health condition or substance use disorder. Furthermore, ongoing monitoring of ART adherence and discontinuation in the Medicare population will be needed given the anticipated growth in the number of Medicare beneficiaries with HIV expected to occur in the coming decade.

### Electronic Supplementary Material

Below is the link to the electronic supplementary material.

Supplementary Material 1
