# Supplementary material for: Antiretroviral Treatment Gaps and Adherence Among People with HIV in the U.S. Medicare Program
Source: AIDS Behav. 2023 Oct 27;28(3):1002–14. doi: 10.1007/s10461-023-04208-8 (PMC10896863; doi:10.1007/s10461-023-04208-8)
Supplement: Supplementary file 1 — Supplementary Material 1 [file 10461_2023_4208_MOESM1_ESM.docx]

**Appendix Table 1. FDA approved Anchor HIV Antiretroviral Therapies**

| Product | Trade name | Acronyms | Class |
| --- | --- | --- | --- |
| **Non-Nucleoside Reverse Transcriptase Inhibitors (NNRTIs)** | | |  |
| Delavirdine | Rescriptor^®^ | DLV | NNRTI |
| Efavirenz | Sustiva^®^ | EFV | NNRTI |
| Etravirine | Intelence | ETR | NNRTI |
| Nevirapine | Viramune^®^ XR | NVP | NNRTI |
| Rilpivirine | Edurant^®^ | RPV | NNRTI |
| **Protease Inhibitors (PIs)** | | | |
| Amprenavir | Agenerase^®^ | APV | PI |
| Atazanavir | Reyataz^®^ | ATV | PI |
| Fosamprenavir | Lexiva^®^ | FOS-APV, FPV | PI |
| Tipranavir | Aptivus^®^ | TPV | PI |
| Indinavir | Crixivan^®^ | IDV | PI |
| Darunavir | Prezista^®^ | DRV | PI |
| Saquinavir mesylate | Invirase^®^ | SQV | PI |
| Nelfinavir mesylate | Viracept^®^ | NFV | PI |
|  |  |  |  |
| **Integrase Strand Transfer Inhibitors (INSTIs)** | | | |
| Dolutegravir | Tivicay^®^ | DTG | INSTI |
| Raltegravir | Isentress^®^ | RAL | INSTI |
| Elvitegravir | Vitekta^®^ | EVG | INSTI |
| **Protease Inhibitor Combinations** | | | |
| Lopinavir/ritonavir | Kaletra^®^ | rLPV | PI/Boost |
| Atazanavir/cobicistat | Evotaz^®^ | cATV | PI/Boost |
| Darunavir/cobicistat | Prezcobix^®^ | cDRV | PI/Boost |
| **Single Tablet Regimen Combinations (STRs)** | | | |
| [Dolutegravir/rilpivirine](https://aidsinfo.nih.gov/drugs/588/juluca/0/patient/) | Juluca^®^ | DTG/RPV | INSTI/NNRTI |
| Dolutegravir/lamivudine | Dovato^®^ | DTG/3TC | INSTI/NRTI |
| [efavirenz/emtricitabine/tenofovir](https://aidsinfo.nih.gov/drugs/424/atripla/0/patient) DF | Atripla^®^ | EFV/FTC/TDF | NNRTI/NRTI/NRTI |
| [efavirenz/lamivudine/tenofovir](https://aidsinfo.nih.gov/drugs/594/symfi/0/patient/) DF | Symfi^®^ | EFV/3TC/TDF | NNRTI/NRTI/NRTI |
| [efavirenz/lamivudine/tenofovir](https://aidsinfo.nih.gov/drugs/592/symfi-lo/0/patient/) DF | Symfi Lo^®^ | EFV/3TC/TDF | NNRTI/NRTI/NRTI |
| [rilpivirine/emtricitabine/tenofovir](https://aidsinfo.nih.gov/drugs/558/odefsey/0/patient) alafenamide | Odefsey^®^ | RPV/FTC/TAF | NNRTI/NRTI/NRTI |
| [rilpivirine/emtricitabine/tenofovir](https://aidsinfo.nih.gov/drugs/441/complera/0/patient) DF | Complera^®^ | RPV/FTC/TDF | NNRTI/NRTI/NRTI |
| Darunavir/cobicistat/emtricitabine/tenofovir alafenamide | Symtuza^®^ | cDRV/FTC/TAF | PI/Boost/NRTI/NRTI |
| [dolutegravir/abacavir/lamivudine](http://aidsinfo.nih.gov/drugs/534/triumeq/0/patient) | Triumeq^®^ | DTG/ABC/3TC | INSTI/NRTI/NRTI |
| [bictegravir/emtricitabine/tenofovir alafenamide](https://aidsinfo.nih.gov/drugs/589/biktarvy/0/patient/) | Biktarvy^®^ | BIC/FTC/TAF | INSTI/NRTI/NRTI |
| [elvitegravir/cobicistat/emtricitabine/tenofovir alafenamide](https://aidsinfo.nih.gov/drugs/553/genvoya/0/patient) | Genvoya^®^ | cEVG/FTC/TAF | INSTI/Boost/NRTI/NRTI |
| [elvitegravir/cobicistat/emtricitabine/tenofovir DF](https://aidsinfo.nih.gov/drugs/507/stribild/0/patient) | Stribild^®^ | cEVG/FTC/TDF | INSTI/Boost/NRTI/NRTI |

**Appendix Table 2. Sample attrition**

| Sample selection criteria | N | % |
| --- | --- | --- |
| All people with any anchor* ART filled between January 1, 2014 to December 31, 2017 | 172,082 | 100.0% |
| Identification group 1: People who did not have any anchor ART fill during the 12-month period before first anchor ART prescription fill between January 1, 2014 to December 31. The first anchor ART prescription was the index agent and the first filled date was the index date. | 58,208 | 33.8% |
| Identification group 2: People who did not qualify for Group 1 and switched the anchor ART agent after the first anchor ART prescription fill between January 1, 2014 to December 31, 2017. The first switch date was the index date for this second group and the switch to agent was the index agent | 66,509 | 38.6% |
| Final Identified Group: Combine identification group 1 and identification group 2 | 124,717 | 72.5% |
| People with continuous eligibility for fee-for-service Medicare Parts A and B in the 12-month pre-index period | 65,458 | 52.5% |
| People with continuous stand-alone Part D coverage in the 12-month pre-index period | 58,164 | 88.9% |
| People with continuous eligibility for fee-for-service Medicare Parts A and B in the 12-month post-index period or continuous eligibility until death during 12-month post-index period | 52,871 | 90.9% |
| People with continuous stand-alone Part D coverage in the 12-month post-index or continuous eligibility until death during 12-month post-index period | 51,551 | 97.5% |
| People with ≥1 inpatient or outpatient claim with an HIV diagnosis (ICD-9-CM codes 042, V08, 079.53 or ICD-10-CM codes B20, B97.35, O98.711, O98.712, O98.713, O98.719, O98.72, O98.73, Z21) on the index date or in the 12-months pre-index period | 50,868 | 98.7% |
| People were alive during 12-months follow up period | 48,641 | 95.6% |
| People do not have missing value for key covariates (i.e., age, sex, identification code for county of residence) | 48,641 | 100.0% |
| People who started only one single agent from index anchor ART class (e.g., exclude those who started 2+ INSTIs) | 48,627 | 99.9% |

**Appendix Table 3. Stratified Analyses to Examine Association of Index Anchor ART Medication Class and STR status with Adherence and Discontinuation**

|  | **Multinomial^a^ logit models^b^** | | | | | | | | | | | | | | | | | | | | | | | | | | | | |  | | **Binomial logit models^b^** | | | | | | | | | |
| --- | --- | --- | --- | --- | --- | --- | --- | --- | --- | --- | --- | --- | --- | --- | --- | --- | --- | --- | --- | --- | --- | --- | --- | --- | --- | --- | --- | --- | --- | --- | --- | --- | --- | --- | --- | --- | --- | --- | --- | --- | --- |
|  | **PDC >=0.70 to <0.95 vs PDC>=0.95** | | | | | | | | | | |  | | | | **PDC <0.70 vs PDC>=0.95** | | | | | | | | | | | | | |  | | **Discontinuation (90-day gap)** | | | | | | | | | |
|  | OR | | 95% CI | | | Chi-Square | | | P value | | | |  | | | | OR | | | 95% CI | | | | | Chi-Square | | P value | | |  | OR | | | 95% CI | | | Chi-Square | | P value | |  |
| **Subgroup of INSTI-based regimen users** | |  | |  |  | | |  | | |  | | | |  | | | |  | | |  | |  | | | |  | |  | |  |  | |  | | |  | |  | |
| STR | 0.88 | | 0.83 | 0.94 | | | 15.28 | | | <.0001 | | | |  | | | | 0.71 | | | 0.65 | | 0.77 | | | 67.92 | | | <.0001 |  | 0.71 | | | 0.64 | | 0.78 | 51.21 | | <.0001 | | |
| MTR (Reference) | |  | |  |  | | |  | | |  | | | |  | | | |  | | |  | |  | | | |  | |  | |  |  | |  | | |  | |  | |
| **Subgroup of NNRTI-based regimen users** | |  | |  |  | | |  | | |  | | | |  | | | |  | | |  | |  | | | |  | |  | |  |  | |  | | |  | |  | |
| STR | 0.76 | | 0.66 | 0.87 | | | 16.55 | | | <.0001 | | | |  | | | | 0.58 | | | 0.48 | | 0.69 | | | 35.51 | | | <.0001 |  | 0.75 | | | 0.62 | | 0.92 | 8.09 | | 0.005 | | |
| MTR (Reference) | |  | |  |  | | |  | | |  | | | |  | | | |  | | |  | |  | | | |  | |  | |  |  | |  | | |  | |  | |
| **Subgroup of MTR users** | |  | |  |  | | |  | | |  | | | |  | | | |  | | |  | |  | | | |  | |  | |  |  | |  | | |  | |  | |
| INSTI | 0.65 | | 0.51 | 0.84 | | | 11.43 | | | 0.001 | | | |  | | | | 0.62 | | | 0.44 | | 0.87 | | | 7.36 | | | 0.007 |  | 0.79 | | | 0.72 | | 0.87 | 25.07 | | <.0001 | | |
| NNRTI | 0.67 | | 0.52 | 0.86 | | | 9.48 | | | 0.002 | | | |  | | | | 0.63 | | | 0.44 | | 0.90 | | | 6.46 | | | 0.011 |  | 0.91 | | | 0.80 | | 1.03 | 2.18 | | 0.140 | | |
| PI (Reference) | |  | |  |  | | |  | | |  | | | |  | | | |  | | |  | |  | | | |  | |  | |  |  | |  | | |  | |  | |
| **Subgroup of STR users** | |  | |  |  | | |  | | |  | | | |  | | | |  | | |  | |  | | | |  | |  | |  |  | |  | | |  | |  | |
| INSTI | 0.97 | | 0.90 | 1.05 | | | 0.45 | | | 0.503 | | | |  | | | | 0.98 | | | 0.89 | | 1.09 | | | 0.13 | | | 0.717 |  | 0.82 | | | 0.74 | | 0.92 | 11.26 | | 0.001 | | |
| NNRTI (Reference) | |  | |  |  | | |  | | |  | | | |  | | | |  | | |  | |  | | | |  | |  | |  |  | |  | | |  | |  | |
| PI | | NA^c^ | |  |  | | |  | | |  | | | | NA^c^ | | | |  | | |  | |  | | | |  | |  | | NA^c^ |  | |  | | |  | |  | |

Abbreviations: ART: antiretroviral therapy; CI: confidence interval; INSTI: Integrase Strand Transfer Inhibitor; MTR: multi-tablet regimen; NNRTI: Non-Nucleoside Reverse Transcriptase Inhibitor; OR: odds ratio; PDC: proportion of days covered; PI: Protease Inhibitor; STR: single-tablet regimen.

^a^Multinomial logit on three categories of outcomes (PDC <0.70 and 0.70≤ PDC <0.95 versus PDC ≥0.95)

^b^Regression models adjusted for all other covariates shown in Tables 3 and 4 of manuscript

^c^Since PIs were not available as a fixed-dose STR until 2018 (i.e. until after our sample identification window).

Note: Subgroup of PI-based regimen users not shown since PIs did not have STR formulation until 2018 (i.e. until after the study sample identification window).
